# HTRecNet: a deep learning study for efficient and accurate diagnosis of hepatocellular carcinoma and cholangiocarcinoma

**DOI:** 10.3389/fcell.2025.1549811

**Published:** 2025-03-24

**Authors:** Jingze Li, Yupeng Niu, Junwu Du, Jiani Wu, Weichen Guo, Yujie Wang, Jian Wang, Jiong Mu

**Affiliations:** ^1^ College of Information Engineering, Sichuan Agricultural University, Ya’ an, China; ^2^ Artificial Intelligence Laboratory, Sichuan Agricultural University, Ya’ an, China; ^3^ Department of Hepatobiliary Pancreaticosplenic Surgery, Ya ‘an People’s Hospital, Ya’ an, China; ^4^ Department of Neurology, Ya’an People’s Hospital, Ya’ an, China; ^5^ Department of Neurology, The Affiliated Hospital, Southwest Medical University, Luzhou, China

**Keywords:** hepatocellular carcinoma (HCC), cholangiocarcinoma (CCA), deep learning, histopathological analysis, automated diagnosis

## Abstract

**Background:**

Hepatocellular carcinoma (HCC) and cholangiocarcinoma (CCA) represent the primary liver cancer types. Traditional diagnostic techniques, reliant on radiologist interpretation, are both time-intensive and often inadequate for detecting the less prevalent CCA. There is an emergent need to explore automated diagnostic methods using deep learning to address these challenges.

**Methods:**

This study introduces HTRecNet, a novel deep learning framework for enhanced diagnostic precision and efficiency. The model incorporates sophisticated data augmentation strategies to optimize feature extraction, ensuring robust performance even with constrained sample sizes. A comprehensive dataset of 5,432 histopathological images was divided into 5,096 for training and validation, and 336 for external testing. Evaluation was conducted using five-fold cross-validation and external validation, applying metrics such as accuracy, area under the receiver operating characteristic curve (AUC), and Matthews correlation coefficient (MCC) against established clinical benchmarks.

**Results:**

The training and validation cohorts comprised 1,536 images of normal liver tissue, 3,380 of HCC, and 180 of CCA. HTRecNet showed exceptional efficacy, consistently achieving AUC values over 0.99 across all categories. In external testing, the model reached an accuracy of 0.97 and an MCC of 0.95, affirming its reliability in distinguishing between normal, HCC, and CCA tissues.

**Conclusion:**

HTRecNet markedly enhances the capability for early and accurate differentiation of HCC and CCA from normal liver tissues. Its high diagnostic accuracy and efficiency position it as an invaluable tool in clinical settings, potentially transforming liver cancer diagnostic protocols. This system offers substantial support for refining diagnostic workflows in healthcare environments focused on liver malignancies.

## 1 Introduction

Liver cancer stands as a significant contributor to global mortality from cancer (McGlynn et al., 2024; Oh and June 2023), with Hepatocellular Carcinoma (HCC) and Cholangiocarcinoma (CCA) emerging as the predominant forms of primary liver cancer (Choi and Thung, 2024; Gurzu et al., 2024). HCC arises from hepatocytes and constitutes the majority (70%–85%) of liver cancer cases, often linked to chronic liver conditions such as hepatitis B or C infections, cirrhosis (Kinsey and Lee, 2024; Rich, 2024; Mak et al., 2024; Wang and Deng, 2023), among others. Conversely, CCA, originating from epithelial cells within the bile ducts, encompasses intrahepatic and extrahepatic cholangiocarcinoma subtypes (Moris et al., 2023; Yang and Zhang, 2023). Despite its lower incidence, timely and precise diagnosis of CCA is critical for enhancing patient outcomes.

Presently, liver cancer diagnosis heavily relies on radiologists’ visual interpretation of imaging scans. However, this approach is prone to subjectivity, potentially leading to misdiagnosis or overlooked cases (Singh et al., 2023; Chatzipanagiotou et al., 2024). Moreover, conventional image analysis techniques exhibit limited sensitivity and specificity, particularly in early-stage liver cancer detection and the identification of rare CCA variants, posing significant challenges (Bakrania et al., 2023). Thus, the imperative lies in developing an efficient, accurate, and automated diagnostic solution to enhance early detection rates and diagnostic precision in liver cancer cases.

In recent years, the application of artificial intelligence (AI) technology, particularly Deep Learning (DL) (Nazir et al., 2024; Rai et al., 2024), in medical image analysis has revolutionized liver cancer diagnosis. Convolutional Neural Networks (CNNs), a pivotal component of DL, exhibit remarkable capabilities in image analysis (Iqbal et al., 2023). By autonomously extracting intricate visual patterns and structural details from original images through multi-level feature learning, CNNs can identify disease-specific features. Studies have demonstrated CNNs’ superiority in tumor diagnosis (Lakshmipriya et al., 2023), cardiovascular disease (Lopez et al., 2023), and other domains compared to human experts, offering a more objective and precise foundation for clinical decision-making. Nevertheless, existing classification methods for Hepatocellular Carcinoma (HCC) and Cholangiocarcinoma (CCA) suffer from drawbacks like high model complexity and demanding computational resources.

**FIGURE 1:**
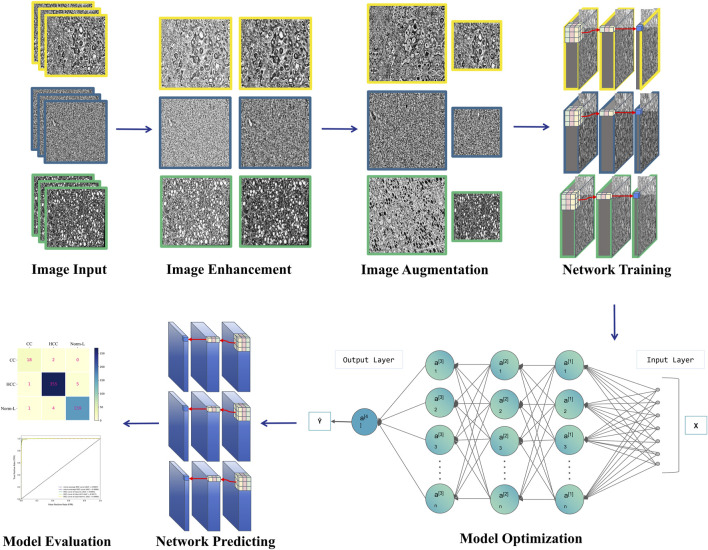
Workflow diagram.

Given the significance of early liver cancer diagnosis and the challenges in image interpretation, there’s a growing focus on developing objective, efficient, and accurate deep learning-based diagnostic tools. These tools aim to automatically detect subtle pathological changes in medical images using techniques such as CNNs, thereby reducing reliance on operator expertise, enhancing diagnostic accuracy and consistency, and facilitating early identification and intervention for liver cancer to improve patient outcomes. This study endeavors to devise a deep learning model for the automatic differentiation of HCC, CCA, and Normal Liver Tissue (Norm-L). By employing innovative model design and optimized image enhancement techniques, this research aims to overcome the limitations of individual models and enhance diagnostic accuracy and generalization capabilities [Figure 1].

Specifically, the innovations of this study include.

(1) Model Design Innovation: This study pioneers the development of an efficient CNN model tailored specifically for the recognition of liver and its associated structural changes. The aim is to enhance the model’s sensitivity and specificity towards Hepatocellular Carcinoma (HCC) and Cholangiocarcinoma (CCA). The model design intricately focuses on optimizing both structural and feature extraction aspects to better accommodate feature expression and facilitate accurate recognition of these cancer types.
(2) Image Enhancement and Optimization: Leveraging advanced image enhancement and image broadening techniques, this study enhances the model’s capability to discern minute pathological changes within the images (Gong et al., 2024; Mumuni and Mumuni, 2022). These techniques not only improve the model’s performance but also ensure its stability across images of varying qualities.
(3) Model Validation Rigor: To ascertain the robustness and generalizability of the proposed model, a rigorous validation strategy is implemented. This includes comprehensive five-fold cross-validation and independent external test set validation (AG et al., 2024; Hassan et al., 2024). Such meticulous validation procedures ensure the stability of the model across diverse datasets, further substantiating its reliability and potential for clinical deployment.
(4) Heat Map Visualization: Employing heat map visualization techniques, this study elucidates the feature extraction outcomes of the model across different types of liver cancer. By offering interpretability of the diagnostic process, these visualizations enhance clinicians’ trust and comprehension of the model’s functionality (Gu, 2022; Nazir et al., 2023).

In summary, this study endeavors to markedly enhance the accuracy and efficiency of early liver cancer diagnosis through groundbreaking deep learning methodologies. By furnishing clinicians with more dependable diagnostic tools, it aims to significantly improve patient outcomes.

## 2 Materials and methods

### 2.1 Data collection

The liver tumor classification dataset utilized in this study was sourced from the Roboflow Universe platform, comprising 192 images of Cholangiocarcinoma (CCA), 3,604 images of Hepatocellular Carcinoma (HCC), and 1,636 images of normal liver tissue (Norm-L) (Gowtham, 2024). This dataset was divided into two parts: a training and validation set, and an external independent testing set. Specifically, 180 CCA images, 3380 HCC images, and 1,536 Norm-L images were used for training and validation, while the external independent testing set consisted of 12 CCA images, 224 HCC images, and 100 Norm-L images, totaling 336 images. The external independent testing set was selected from the same overall dataset but was strictly separated from the training and validation set to ensure unbiased evaluation of the model’s generalization capability. This separation strategy was designed to accurately assess the model’s performance on unseen data, simulating real-world diagnostic scenarios more effectively.

To ensure heterogeneity and minimize the risk of overfitting, the images were obtained from different liver samples from multiple patients. Specifically, the HCC and CCA images were sourced from distinct liver samples from different patients, while the Norm-L images were obtained from both varied regions of the same liver and different healthy donors, offering a comprehensive representation of normal liver morphology. All images were acquired using the Aperio AT2 high-resolution digital pathology scanner at 20× to ×40 magnification with a resolution of approximately 0.25 µm per pixel, ensuring sufficient detail for robust feature extraction. The images were stained using a standard Hematoxylin and Eosin (HE) staining protocol, which provides clear contrast between cellular structures.

Notably, there exists a significant data imbalance within this dataset, particularly evident in the limited number of CCA samples. To address this challenge, a series of image enhancement and augmentation techniques, such as rotation, scaling, and panning, were employed during data preprocessing (Maharana et al., 2022). These techniques aimed to enhance data diversity and facilitate more robust model training. Moreover, the high-resolution images underwent standardization to ensure uniformity and reliability of the data (Seoni et al., 2024). Each image was meticulously annotated by domain experts to ensure accuracy. The diversity and superior quality of this dataset render it well-suited for supporting automated classification studies of liver cancer.

### 2.2 Data preparation

In this study, meticulous preprocessing and enhancement of the collected raw data were undertaken to optimize the feature recognition and classification capabilities of hepatocyte images. Initially, to ensure consistency and computational efficiency of model inputs, all images were uniformly resized to a standard size of 224 × 224 pixels, which refers to the fixed input dimensions required by the convolutional neural network rather than the physical resolution of the images. This resizing operation ensures that images of different original dimensions can be processed in a unified manner without affecting their diagnostic features. Additionally, image augmentation techniques, including random cropping, were applied before resizing. Random cropping was performed on original images to generate different feature variations, after which the cropped regions were resized to 224 × 224 pixels. This ensures that augmented samples maintain compatibility with the model’s input requirements while enhancing feature diversity and model robustness.

**FIGURE 2:**
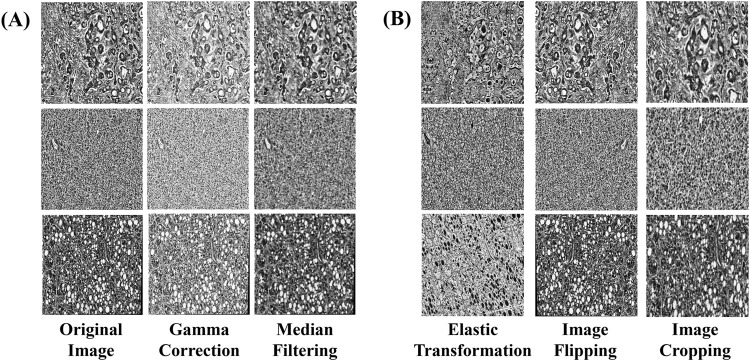
Examples of data augmentation and augmentation techniques. **(A)** Image enhancement: The image processed by gamma correction and median filtering, **(B)** Image augmentation: images processed through elastic transformation, horizontal and vertical flipping, and random cropping.

To further augment the learning performance of the model, a series of advanced data enhancement techniques were employed to enhance the diversity and robustness of the training set. Gamma correction was applied to adjust brightness and contrast, ensuring that details in both dark and bright regions remained distinguishable (Dash et al., 2023). To prevent excessive contrast reduction, the gamma value was selected based on the image intensity distribution, maintaining a balance between enhancement and preservation of diagnostic features. This adjustment facilitated the recognition of fine pathological details, contributing to improved sensitivity and specificity. Median filtering was used to suppress random noise, particularly fine texture noise in histopathological images (Samanta et al., 2023). A kernel size of 3 × 3 was employed to ensure noise reduction while preserving critical tissue structures, minimizing the risk of texture loss. Empirical evaluation confirmed that this approach effectively enhanced image quality without compromising diagnostic information. To address concerns regarding smooth shading, enhancement parameters were fine-tuned to prevent over-smoothing while maintaining the visibility of essential pathological structures. The combination of gamma correction and median filtering was optimized to retain feature integrity, ensuring that diagnostic elements remained distinct throughout the preprocessing pipeline. The application of these techniques is illustrated in (Figure 2A).

In this study, additional image augmentation techniques including elastic transform, horizontal and vertical flipping, and random cropping were employed to further diversify the data samples and replicate various shooting conditions and perspectives. Each training image underwent augmentation multiple times with randomized transformations, ensuring that the model encountered the same base image in different augmented forms across different epochs. This approach maximized data diversity without overfitting, as no two epochs presented the exact same augmented images.

Elastic Transform applies elastic deformation to the image, simulating different levels of stretching and compression. This allows the model to learn image features under varying deformation conditions, thereby enhancing its generalization capability. Horizontal and Vertical Flip involves flipping the image in different directions to increase diversity and prevent bias during model training. Random Cropping selects random regions within the image for cropping, ensuring the model’s robustness to positional variation and enabling better generalization across different viewpoints and in the presence of occlusions. The implementation of these augmentation and broadening techniques is illustrated in (Figure 2B).

The combination of these data enhancement methods not only enhances image quality but also improves the model’s ability to identify features associated with Hepatocellular Carcinoma and Cholangiocarcinoma. This establishes a robust foundation for the model’s generalization and diagnostic accuracy. These meticulous data preparation steps ensure the model’s proficiency in extracting crucial information from hepatocyte images, providing an efficient and reliable tool for the automated diagnosis of liver cancer.

### 2.3 Model construction

This study aims to balance model complexity and accuracy to effectively address the demands for efficiency and precision in liver cancer diagnosis. ResNet50 was selected as the benchmark model due to its well-established effectiveness in medical image classification tasks (Pisarcik et al., 2024). With its deep hierarchical structure and residual connections, ResNet50 provides a robust framework for feature extraction while mitigating vanishing gradient issues, making it a suitable choice for histopathological image analysis.

However, standard ResNet50 lacks specialized mechanisms for handling fine-grained pathological variations in HCC and CCA. To enhance the model’s capability in distinguishing these liver cancer subtypes, two complementary modifications were introduced: SPConv and CBAM. SPConv improves computational efficiency by reducing redundant feature computation while preserving essential information, which is particularly beneficial given the high-resolution and complex nature of histopathological images. CBAM enhances feature selection by introducing spatial and channel attention, allowing the model to focus on diagnostically relevant regions. The combination of these two modules provides a balance between efficiency and accuracy, addressing both computational constraints and feature discrimination challenges specific to liver cancer classification.

#### 2.3.1 Improvement A: ResSPNet

**FIGURE 3:**
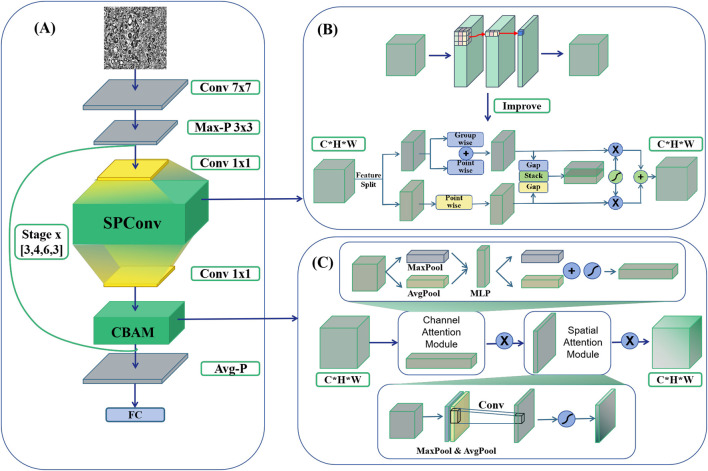
HTRecNet architecture diagram. **(A)** The backbone of HTRecNet based on ResNet50. **(B)** Illustrates the integration of the SPConv module, which replaces the conventional 3 × 3 convolution in residual blocks with a split-based feature processing mechanism. **(C)** Depicts the incorporation of the CBAM attention mechanism, positioned immediately before the residual connection.

The initial enhancement in this study involves integrating the SPConv module into the benchmark model to further refine its performance. SPConv (Split-based Convolution) is employed to reduce computational complexity while upholding model efficacy. This is achieved by partitioning the input feature map into two components: a representative part and a redundant part. These components undergo separate convolution operations, thereby decreasing computational complexity while preserving model performance (Wang et al., 2021). The representative part utilizes a traditional 3 × 3 convolutional kernel for feature extraction, whereas the redundant part employs a lightweight 1 × 1 convolutional kernel to address subtle feature distinctions (Figure 3A).

The SPConv module is specifically devised to tackle the issue of redundancy within feature map patterns inherent in convolutional operations. Research indicates that numerous feature maps exhibit considerable similarity in pattern, thus redundancy can be mitigated by retaining representative features and employing less computational resources to handle redundant features. Through this split-fusion approach, the SPConv module effectively diminishes parameters and computation, while simultaneously maintaining or enhancing model accuracy.

The SPConv module initially divided the input channels into two segments: a representative part and a redundant part. The representative segment underwent feature transformation through a 3 × 3 convolutional kernel to extract intrinsic information, while the redundant segment was processed by a lightweight 1 × 1 convolutional kernel to capture subtle complementary details. To integrate these two feature types effectively, a parameter-free feature fusion module was employed. Instead of simple concatenation or element-wise addition, this fusion mechanism leveraged global average pooling (GAP) to extract channel-wise statistics, followed by a soft attention operation to compute feature importance weights. The final fused representation was obtained through a weighted sum of the representative and redundant features, allowing the network to dynamically adjust the contribution of each feature type to enhance information representation and model expressiveness.

By integrating the SPConv module into the model, this study effectively maintains diagnostic accuracy while significantly reducing computational complexity. This innovative convolution module design opens up new possibilities for developing efficient and precise liver cancer diagnostic models.

#### 2.3.2 Improvement B: ResCBANet

The study’s second enhancement focuses on enhancing the feature recognition capabilities for Hepatocellular Carcinoma (HCC) and Cholangiocarcinoma (CCA) by proposing an innovative model refinement strategy that integrates the Convolutional Block Attention Module (CBAM) into the baseline model ResNet50 (Clausen et al., 2021). CBAM, known for its lightweight and effective attention mechanism, significantly enhances the representation capabilities of convolutional neural networks (Figure 3B).

The CBAM module executes adaptive feature reconstruction by sequentially deducing attention maps in both channel and spatial dimensions, and subsequently multiplying these maps with the input feature maps. Specifically, CBAM initiates by generating two distinct spatial context descriptors in the channel dimension through average pooling and maximum pooling (Zafar et al., 2022) operations. These descriptors are then channeled through a shared multilayer perceptron (MLP) (Zafar et al., 2022) to compute the channel attention map. Following this, the input feature maps undergo weighting using the channel attention maps to emphasize crucial features while suppressing unnecessary ones.

After generating the channel attention map, CBAM proceeds to generate a spatial attention map. This involves creating two 2D feature maps through average pooling and maximum pooling operations in the channel dimension. These feature maps are then combined in the channel dimension, and a convolutional layer is employed to produce the final spatial attention map. This process aids the network in focusing more effectively on critical regions within the image, thereby enhancing the model’s representation and classification performance.

Incorporating the CBAM module into the benchmark model leads to a significant improvement in the model’s diagnostic accuracy while maintaining low computational complexity. Experimental results demonstrate that the CBAM-enhanced network surpasses the benchmark model in tasks such as image classification and target detection, underscoring its broad applicability and effectiveness. This enhancement holds substantial importance for augmenting the accuracy and efficiency of early liver cancer diagnosis. By integrating the CBAM attention mechanism, the refined model becomes adept at handling intricate features present in liver cancer images, thereby augmenting its capability to identify HCC and CCA. Consequently, this advancement furnishes a more precise and dependable tool for the early diagnosis of liver cancer.

#### 2.3.3 HTRecNet

In this study, a novel and efficient deep learning model, HTRecNet, was devised by introducing structural refinements to the ResNet50 backbone, incorporating two previously mentioned enhancements: the CBAM attention mechanism and the SPConv module (Figure 3). The modifications aim to optimize feature representation and computational efficiency while preserving the residual learning framework of ResNet50, thereby facilitating accurate and efficient classification of Hepatocellular Carcinoma (HCC) and Cholangiocarcinoma (CCA).

To elaborate, HTRecNet restructures the feature transformation pathway within each residual block to enhance information selectivity. The conventional 3 × 3 convolution was replaced with SPConv, which partitions feature processing into representative and redundant components. This structure preserves key discriminative features while reducing unnecessary computations, thereby balancing computational complexity and model expressiveness. Additionally, CBAM was incorporated before the residual connection to refine feature importance through channel and spatial attention, ensuring that diagnostically relevant patterns are emphasized before merging with the residual pathway.

The revised residual block structure follows the sequence: 1 × 1 convolution → 3 × 3 SPConv → 1 × 1 convolution → CBAM → residual connection, maintaining the hierarchical feature extraction benefits of ResNet50 while introducing improved feature calibration and computational efficiency. These refinements collectively enhance HTRecNet’s capability to capture fine-grained pathological features with improved robustness and reduced computational overhead, making it a strong candidate for early diagnosis and precision medicine applications in liver cancer.

### 2.4 Model evaluation

In order to comprehensively evaluate the performance of deep learning models in diagnosing Hepatocellular Carcinoma (HCC) and Cholangiocarcinoma (CCA), a multi-dimensional evaluation system was constructed in this study. The system integrates several key metrics, including Accuracy (ACC), Precision, Recall, F1 Score, Matthews Correlation Coefficient (MCC), Area Under the Curve (AUC), Confusion Matrix, and Floating Point Operations (FLOPs) to thoroughly assess predictive accuracy, classification comprehensiveness, computational complexity, and consistency with actual diagnoses.

Accuracy reflects the overall correctness of the model’s classification and is calculated as:

$$\text{Accuracy}=\frac{\text{TP}+\text{TN}}{\text{TP}+\text{TN}+\text{FP}+\text{FN}}$$

Where TP (True Positive) is a true case, TN (True Negative) is a true negative case, FP (False Positive) is a false positive case, and FN (False Negative) is a false negative case.

Precision and recall are employed to evaluate classification performance, particularly in scenarios where class imbalance exists. For the three-class classification task in this study, these metrics were extended using micro-average and macro-average approaches.

During training, the micro-average approach was used, which aggregates true positives, false positives, and false negatives across all classes before computing overall metrics:

$$Precision_{micro}=\frac{\sum TP}{\sum \left(TP+FP\right)}$$

$${Recall}_{micro}=\frac{\sum TP}{\sum \left(TP+FN\right)}$$

This method provides a global assessment of model performance by considering all classes equally, thus effectively handling class imbalance.

During testing, both micro-average and macro-average methods were employed to ensure a balanced evaluation. The macro-average approach calculates precision and recall for each class independently and then takes the arithmetic mean:

$$Precision_{macro}=\frac{1}{N}\sum_{i=1}^{N}\;\frac{TP_{i}}{TP_{i}+FP_{i}}$$

$$Recall_{macro}=\frac{1}{N}\sum_{i=1}^{N}\;\frac{TP_{i}}{TP_{i}+FN_{i}}$$

where N is the number of classes. The macro-average method treats all classes equally regardless of their sample sizes, offering an overall evaluation of model performance across all categories.

By employing both micro-average and macro-average approaches, this study ensures a comprehensive evaluation of the model’s capability in handling imbalanced data during training and testing phases.

F1 Score is the harmonic mean of precision and recall, making it particularly suitable for handling class imbalance, calculated as:

$$F1\text{Score}=2\times \frac{\text{Precision}\times \text{Recall}}{\text{Precision}+\text{Recall}}$$

A high F1 score shows that the model has a good balance between precision and recall.

The Matthews correlation coefficient (MCC) is a robust categorical quality metric that is particularly suitable for unbalanced data. Its value ranges from −1 to +1, with +1 indicating perfect agreement between predictions and true labels, 0 indicating no correlation between predictions and true labels, similar to random classification, and −1 indicating a perfectly inverse relationship, where every prediction is the exact opposite of the true label. The calculation formula is:

$$\text{MCC}=\frac{\text{TP}\times \text{TN}-\text{FP}\times \text{FN}}{\sqrt{\left(\text{TP}+\text{FP}\right)\left(\text{TP}+\text{FN}\right)\left(\text{TN}+\text{FP}\right)\left(\text{TN}+\text{FN}\right)}}$$

MCC more objectively reflects the model’s performance in unbalanced data.

The area under the curve (AUC), derived from the Receiver Operating Characteristic (ROC) curve, quantifies the overall performance of a classifier across all decision thresholds. A high AUC value, nearing 1, signifies strong discriminative ability among different categories, making it suitable even for three-classification problems. In such cases, separate ROC curves for each category relative to the others can be calculated and combined to assess the model’s overall performance. AUC offers a comprehensive evaluation perspective, reflecting the model’s performance under varying decision thresholds.

The Confusion Matrix (CM) provides a visual representation of the relationship between the model’s predictions and the actual labels, highlighting error patterns through True Positives (TP), True Negatives (TN), False Positives (FP), and False Negatives (FN). CM aids in identifying issues like overfitting, underfitting, and skewing, thus guiding model adjustments and optimizations.

Floating Point Operations (FLOPs) measure the total number of floating point calculations required for a single forward pass through the model. FLOPs provide a quantitative evaluation of the model’s computational cost and efficiency, offering insights into its deployment feasibility on resource-constrained devices. A lower FLOPs value indicates a more efficient model with faster inference times and reduced energy consumption, making it particularly suitable for real-time clinical applications. In this study, FLOPs were calculated using the “ptflops” package in PyTorch, which analyzes each layer’s architecture and operations to accurately compute the floating point operations. This approach ensures consistent and reliable measurement of computational complexity across all models compared.

Through this multidimensional assessment system, the study comprehensively evaluates the model’s performance in diagnosing Hepatocellular Carcinoma and Cholangiocarcinoma, ensuring its practical applicability. This comprehensive assessment approach not only offers an in-depth analysis of the model’s classification performance but also identifies potential issues in real-world application, thus guiding further model refinement.

## 3 Results

### 3.1 Experiments

The experimental setup of this study aims to comprehensively evaluate the performance of the proposed model in liver cancer diagnosis. To ensure the stability and generalization ability of the model, the experiments were evaluated using a five-fold cross-validation combined with an independent external test set. The five-fold cross-validation divides the dataset into five equal parts, four of which are selected for training and one for validation each time. This process is repeated five times, and the average value is taken to evaluate the model performance. The independent external test set is then used for the final performance evaluation to ensure that the model performs well on unknown data.

All models underwent parameter optimization using a combination of random and grid search techniques to determine the best hyperparameter combinations. Hyperparameter optimization includes adjusting parameters such as learning rate, batch size, and weight decay. An Early Stopping mechanism was employed during training to prevent overfitting, and the optimal model parameters were recorded. Each model underwent 100 iterations to ensure convergence. The application of data preprocessing, data enhancement, and image augmentation techniques played a crucial role in the training process. These techniques significantly improved the robustness and generalization of the models, contributing to more accurate and reliable results.

The experiments were conducted on a high-performance computer equipped with NVIDIA GeForce RTX 4090 GPUs, Intel(R) Xeon(R) Gold 6230R CPUs @ 2.10GHz, and 256 GB of RAM. These hardware configurations were chosen to ensure the efficiency and accuracy of model training and evaluation. Python 3.8 was selected as the programming language, and PyTorch 1.9.0 was utilized as the deep learning framework. These specific tool and version choices were made to facilitate smooth model development and experimentation.

With the aforementioned experimental setup and details, this study ensures the robustness and efficiency of the model across different environments and datasets, thereby providing a reliable diagnostic aid for liver cancer.

### 3.2 Comparison experiments

**FIGURE 4:**
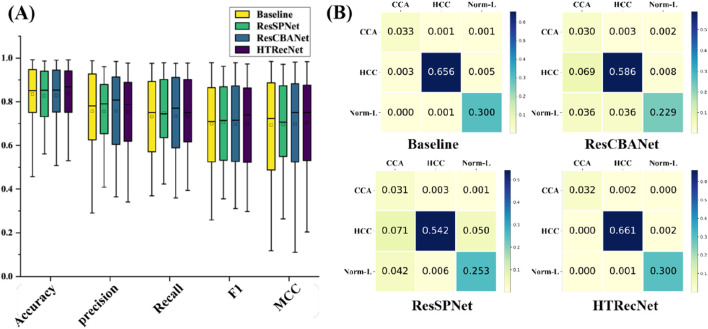
**(A)** Performance distribution: Box plots showing the distribution of key performance metrics across all epochs for each model. **(B)** Confusion matrices: Classification results across the three categories (CCA, HCC, Norm-L) for each model.

In this study, model metrics were compared through ablation experiments to evaluate the performance of different models. The performance and stability of each model across different iteration periods are illustrated in (Figure 4A). Unlike the Baseline model, HTRecNet not only achieves high performance but also demonstrates superior stability and lightweight efficiency by balancing data distribution and high performance. The box plots in [Figure 4A] are generated from the data across all training epochs, representing the overall distribution of model performance rather than a single endpoint value.

The final training results are presented in (Table 1), showcasing the best performance of each model after five-fold cross-validation. HTRecNet achieves the highest scores across all key metrics, with Accuracy (0.98) being 0.01 higher than the Baseline’s 0.97. This indicates that HTRecNet correctly identifies approximately 98 out of every 100 images, compared to 97 for the Baseline. Although this difference may seem small, in real-world clinical scenarios with thousands of samples, even a 1% increase in accuracy can translate to a significantly higher number of correct diagnoses, thus reducing misdiagnosis rates.

**TABLE 1:** Performance comparison of different models.

Model	Accuracy	Precision	Recall	F1	MCC
Baseline	0.98862	0.97227	0.98172	0.97457	0.97593
ResCBANet	0.9912	0.98432	0.97616	0.97875	0.98127
ResSPNet	0.98629	0.9606	0.97819	0.96247	0.97163
**HTRecNet**	**0.99325**	**0.98958**	**0.9837**	**0.98531**	**0.9857**

Bold values indicate the performance metrics of HTRecNet, the proposed method in this study, highlighting its comparative results against other models.

The confusion matrices in (Figure 4B) further illustrate HTRecNet’s advantages, showing a higher number of true positive and true negative cases compared to other models. This confirms the model’s ability to accurately differentiate between categories and highlights its reliability in clinical applications.

**FIGURE 5:**
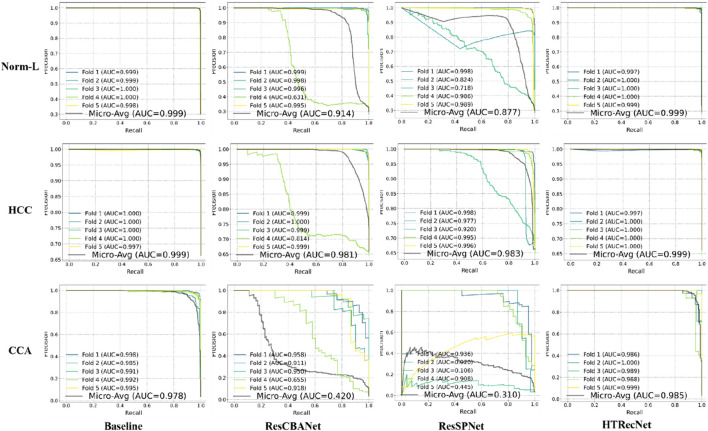
PR curves with micro-average AUC values for each model across the three categories (Norm-L, HCC, CCA).

Additionally, the PR curves in (Figure 5) demonstrate that HTRecNet consistently achieves the highest AUC-PR values across all categories. The model maintains a strong balance between precision and recall, showing enhanced robustness when handling imbalanced class distributions, which ultimately contributes to more reliable diagnostic outcomes.

In summary, HTRecNet demonstrates high accuracy and robust stability across all key performance indicators during five-fold cross-validation. It consistently achieves superior Accuracy, Recall, Precision, F1, MCC and AUC-PR compared to other models, highlighting its precise classification capability. Additionally, the model shows greater stability across different iterations, maintaining consistent performance with minimal fluctuation. These strengths underscore its significant potential and practical application value in liver cancer diagnosis.

HTRecNet has not only demonstrated notable advancements across various metrics such as accuracy but has also exhibited impressive performance concerning model complexity. Upon comparing the model complexity parameters, it becomes evident that HTRecNet surpasses the benchmark model in terms of FLOPs and the number of parameters (Params). The specific comparative results are illustrated in (Table 2), where HTRecNet showcases approximately a 30% reduction in both computational requirements and the number of parameters compared to the benchmark model. This substantial improvement significantly enhances the computational efficiency and resource utilization of the model.

**TABLE 2:** Comparison of complexity of different models.

Model	Params(G)	FLOPs(G)
Baseline	23.51	8.22
ResCBANet	24.46	8.51
ResSPNet	16.31	5.86
HTRecNet	**17.25**	**5.94**

Bold values indicate the performance metrics of HTRecNet, the proposed method in this study, highlighting its comparative results against other models.

In the iterative performance analysis of the models, HTRecNet demonstrates remarkable stability and rapid convergence during training, underscoring its outstanding performance.

**FIGURE 6:**
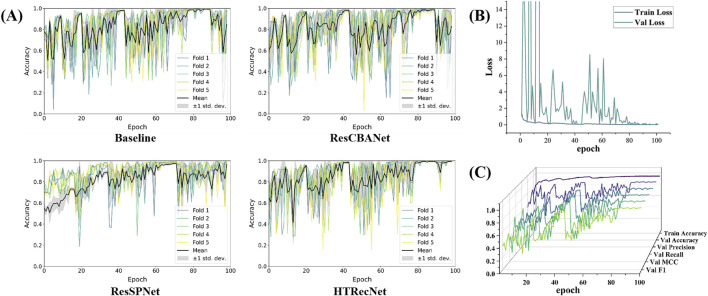
Among them, **(A)** is the accuracy iteration diagram of five fold cross validation for Baseline, ResCBANet, ResSPNet, HTRecNet, **(B)** is the accuracy iteration diagram of various indicators and training for HTRecNet during validation, and **(C)** is the loss iteration diagram of HTRecNet during training and validation.

Firstly, in the iterative plots depicting the five-fold cross-validation accuracy of each model, HTRecNet showcases minimal variance in the later iterations of each fold, indicating consistency across different data subsets. HTRecNet swiftly attains a stable and high level of accuracy in the later iterations while maintaining minimal variance across folds, thereby highlighting its excellent generalization performance across diverse data divisions (Figure 6A).

Secondly, HTRecNet displays a favorable convergence trend in Accuracy, Precision, Recall, F1 Score, and MCC across all key metrics during both training and validation. This trend suggests that the model not only enhances its performance during training but also consistently and steadily optimizes on the validation set. Such behavior serves to verify HTRecNet’s effectiveness and its robust generalization capability (Figure 6B).

Finally, the loss value of HTRecNet gradually decreases and stabilizes in the iterative loss plots of both training and validation. This indicates the model’s effective reduction of prediction error and achievement of a well-fitted state during training, underscoring HTRecNet’s maintenance of efficient learning ability and stable convergence throughout the training and validation process (Figure 6C).

In summary, HTRecNet’s performance during the iterative training process, evidenced by the stability of the five-fold cross-validation accuracy, convergence of various metrics, and loss function, fully demonstrates its superiority in learning efficiency and generalization performance. The model swiftly achieves convergence at different iteration stages and consistently performs on the validation set, thereby proving the robustness and efficiency of HTRecNet in the liver cancer diagnosis task.

### 3.3 External independent testing

Evaluation results on an external independent test dataset demonstrate the outstanding performance of HTRecNet in real-world applications. The model excelled across several metrics, showcasing its potential in liver cancer diagnosis.

**FIGURE 7:**
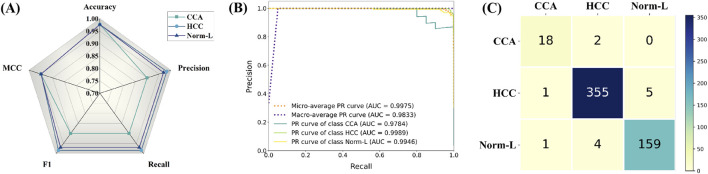
Among them, **(A)** is the radar plots of various indicator data for each category of HTRecNet on an external independent testing dataset, **(B)** is the Precision-Recall (PR) curves of HTRecNet under various categories and their micro and macro average metrics on an external independent testing dataset, and **(C)** is the confusion matrix diagram of HTRecNet on an external independent test dataset.

Firstly, *via* radar plot visualization of data for each index across the three categories of CCA, HCC, and Norm-L, HTRecNet showcased exceptional performance. All indexes within each category surpassed the 90% mark, with many exceeding 95%, Among them, MCC is 0.95 and Accuracy is 0.97 (Figure 7A). This performance underscores the robustness and high accuracy of HTRecNet on external data. Particularly noteworthy is HTRecNet’s strong performance in key metrics such as Accuracy, Precision, Recall, and F1 Score, crucial for practical clinical applications.

Secondly, the Precision-Recall (PR) graphs across the three categories, along with their micro and macro average metrics, further affirm HTRecNet’s excellent performance (Figure 7B). Each AUC-PR value exceeded 0.99, demonstrating the model’s outstanding precision-recall trade-off across different categories. These high AUC-PR values reflect HTRecNet’s capability to maintain high precision and recall simultaneously, effectively distinguishing Hepatocellular Carcinoma (HCC), Cholangiocarcinoma (CCA), and Normal Liver Tissue (Norm-L) even under imbalanced data conditions. This reliable precision-recall balance not only highlights the model’s superior performance in identifying key diagnostic features but also underscores its robustness and consistency in practical applications. By minimizing false positives and missed diagnoses, HTRecNet provides clinicians with accurate and trustworthy decision support, thereby enhancing diagnostic accuracy and reducing clinical risks.

Finally, the confusion matrix diagram clearly illustrates that HTRecNet achieves exceptional classification results across each category (Figure 7C). The matrix indicates very high true-positive and true-negative rates, underscoring the model’s accuracy and reliability in practical scenarios. A high true-positive rate signifies the model’s ability to accurately identify most real cases, while a high true-negative rate demonstrates its effectiveness in excluding non-cases and reducing misdiagnosis. These impressive performance metrics highlight HTRecNet’s potential in liver cancer diagnosis, offering significant support for clinical practice.

In summary, HTRecNet’s performance on the external independent test dataset, reflected in high indicator scores, elevated AUC values in ROC curves, and outstanding confusion matrix performance, underscores its promising role in liver cancer diagnosis. These results not only affirm the validity and reliability of the model but also showcase its practical utility and efficiency in clinical applications.

### 3.4 Heat map visualization

**FIGURE 8:**
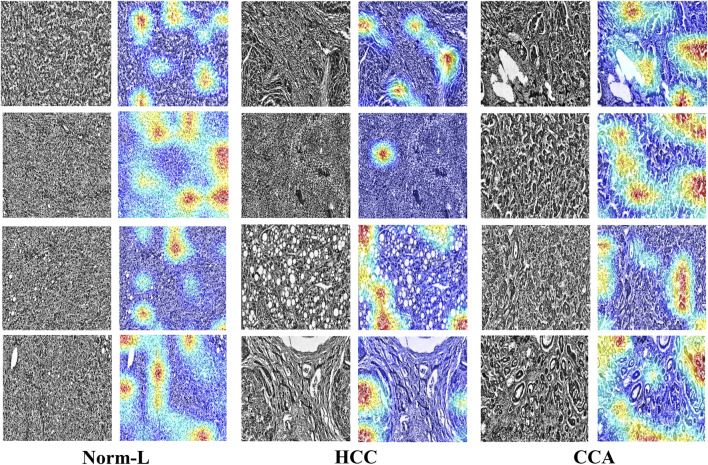
HTRecNet’s feature interest heatmap for CCA, HCC, and Norm-L categories. Each pair of images shows the original pathology image (left) and the corresponding heat map (right), illustrating the model’s attention to key feature regions. Heat maps were generated using Grad-CAM, displaying model attention with a color scheme from blue (low attention) to red (high attention). Each heat map represents a single class detection per image. This comparison demonstrates how HTRecNet utilizes critical features to distinguish between categories, highlighting the model’s decision-making basis.

To further validate HTRecNet’s feature extraction ability in liver cancer diagnosis and enhance the interpretability of the diagnostic process, this study generated heat maps of HTRecNet’s feature interest across three categories: CCA, HCC, and Norm-L (Figure 8). The heat maps were generated using the Grad-CAM (Gradient-weighted Class Activation Mapping) method, which calculates the gradients of the target category relative to the final convolutional layer’s feature maps, highlighting regions that contribute most to the model’s decision. It is important to note that this approach produces a single class detection per image, rather than generating a spatial map for every category within the image.

For CCA, HTRecNet accurately identifies abnormal changes in the bile duct region, showcasing the model’s sensitivity to subtle pathological features. For HCC, the model predominantly highlights structural changes and abnormal proliferation areas of hepatocytes, effectively capturing the typical features of Hepatocellular Carcinoma. Conversely, in Norm-L’s heat map, the model focuses primarily on normal structural regions of the liver, further confirming HTRecNet’s capability to differentiate between normal and diseased liver tissues.

These generated heat maps not only demonstrate HTRecNet’s effectiveness in feature extraction across different categories but also provide high interpretability for the diagnostic process (Talukder et al., 2022; Poursabzi-Sangdeh et al., 2021). By analyzing these heat maps, medical professionals can intuitively grasp the model’s decision-making rationale, thereby increasing trust in the diagnostic outcomes and aiding clinical decision-making.

In conclusion, HTRecNet’s performance in heat map visualization underscores its robust feature extraction capability and efficient classification performance in liver cancer diagnosis, offering substantial technical support for clinical applications.

## 4 Discussion

### 4.1 Research overview

The objective of this study is to develop an efficient and accurate deep learning model, HTRecNet, to aid in the early diagnosis of liver cancer. The study incorporates innovative enhancements to the ResNet50 benchmark model by integrating the SPConv module and the CBAM attention mechanism. Experimental findings demonstrate that HTRecNet excels in classifying HCC, CCA, and normal liver tissues, showcasing exceptional generalization ability and robustness on external independent test datasets. Additionally, the model’s feature extraction capability and interpretability are validated through heat map visualization.

### 4.2 Novelty and importance

The novelty of this study lies in the development of HTRecNet, a deep learning framework that incorporates advanced image processing techniques and model enhancements to improve performance in liver cancer diagnosis. The introduction of the SPConv module and the CBAM attention mechanism addresses key challenges in computational efficiency, feature extraction, and interpretability. The SPConv module reduces computational complexity through a split-fusion strategy, which partitions input features into representative and redundant components. This design minimizes redundant computations while retaining essential feature information, enabling efficient model operation without compromising accuracy.

The CBAM attention mechanism enhances the model’s ability to focus on important pathological features by sequentially applying channel and spatial attention. This mechanism improves feature extraction by emphasizing critical regions in histopathological images, such as hepatocyte abnormalities in HCC and bile duct changes in CCA. The integration of SPConv and CBAM improves classification accuracy and enables robust performance across datasets, particularly in scenarios with imbalanced and limited samples.

Additionally, a comprehensive image preprocessing workflow is implemented, incorporating data enhancement and augmentation techniques to address the imbalance in the dataset, particularly for CCA cases. These workflows contribute to improving the model’s generalization capability, ensuring consistent performance across training and external test datasets. The proposed approach achieves AUC values above 0.99 and an external test accuracy of 97%, demonstrating its ability to differentiate between liver cancer subtypes and normal liver tissues with high reliability.

To enhance clinical utility, the model provides heat map visualizations that offer insights into the regions prioritized during diagnosis, facilitating transparency in the prediction process. This feature supports the interpretability of HTRecNet, making it a useful tool for assisting clinical workflows.

Overall, HTRecNet integrates computational efficiency, accurate feature extraction, and interpretability, offering a methodologically sound approach for early and accurate diagnosis of liver cancers such as HCC and CCA.

### 4.3 Clinical significance

The development of HTRecNet provides an efficient and accurate approach to early liver cancer diagnosis, addressing the clinical need for reliable tools to identify Hepatocellular Carcinoma (HCC) and Cholangiocarcinoma (CCA) at their early stages. The model’s ability to accurately classify liver cancer subtypes and normal liver tissues with an external test accuracy of 97% reduces the risk of misdiagnosis and missed diagnoses, which are significant challenges in current clinical practice. By incorporating advanced image processing techniques and a carefully designed workflow, HTRecNet ensures robust performance even with imbalanced datasets, particularly for the less common CCA cases. This capability enhances its potential utility in a wide range of clinical settings.

The interpretability of HTRecNet is strengthened through the use of heat map visualizations, which provide clear insights into the regions of histopathological images that are prioritized during diagnosis. This transparency not only enables clinicians to understand the model’s decision-making process but also fosters trust in the outcomes of AI-assisted diagnostic tools. Such features make HTRecNet suitable for integration into existing diagnostic workflows, supporting clinicians in confirming diagnoses and streamlining decision-making processes.

Additionally, the efficient design of HTRecNet, achieved through the integration of the SPConv module and the CBAM attention mechanism, ensures that the model operates effectively even in resource-constrained environments, such as smaller clinics or remote healthcare facilities. This makes the model adaptable to diverse healthcare settings, broadening its potential impact on liver cancer diagnosis and patient outcomes.

### 4.4 Limitations

Despite the significant progress made in liver cancer diagnosis in this study, there are still some limitations that need to be addressed in future research.

#### 4.4.1 Dataset imbalance

The dataset in this study contained significantly fewer CCA samples compared to HCC and Norm-L samples. Despite employing various data enhancement and image augmentation techniques to mitigate this issue, the sample imbalance may still limit the model’s ability to generalize for CCA recognition. Specifically, data imbalance can cause the model to favor more common categories, leading to poorer performance in predicting rare categories such as CCA. Future studies should focus on collecting more CCA samples to balance the dataset and enhance the model’s ability to identify rare types of liver cancer.

#### 4.4.2 Limited external validation dataset

While this study demonstrated good performance on external independent test datasets, the number and diversity of these datasets remain limited. External validation is crucial for assessing the model’s generalization ability and its effectiveness in real-world applications. The restricted diversity and quantity of the datasets used in this study may impact the overall evaluation of the model’s performance. Therefore, future studies should conduct validation on larger and multi-center datasets to ensure the stability and reliability of the model across different clinical settings.

#### 4.4.3 Model complexity

Although HTRecNet has been optimized in terms of computational complexity through the introduction of SPConv, its computational resource requirements remain high. This can pose a challenge in resource-constrained environments, particularly in primary care settings or where equipment performance is limited. Further optimization of the model structure to reduce its computational resource requirements, thereby enabling efficient operation even in low-resource environments, will be a crucial area for future research.

#### 4.4.4 Practical challenges in clinical application

There are numerous challenges associated with integrating AI models into clinical practice. These include physicians’ acceptance of AI technology, the complexity of integrating models with existing healthcare processes, and concerns regarding trust in the model decision-making process. While heat map visualization offers some level of interpretability, additional research and practical implementation are necessary to enhance the transparency and interpretability of the model further. This will facilitate widespread acceptance and trust among clinicians.

### 4.5 Future prospects

Future research will aim to enhance the performance and application value of HTRecNet by increasing the number of CCA samples to balance the dataset and improve the model’s generalization ability. Validating HTRecNet’s performance on multi-center datasets will ensure its stability and reliability across various environments. Additionally, efforts will continue to optimize the model structure to reduce computational resource requirements, enabling efficient operation in low-resource settings. Enhancing the interpretability of the model is crucial. Developing more intuitive tools will help clinicians better understand and trust the decision-making process of HTRecNet. Exploring the effectiveness and potential of HTRecNet in real-world diagnosis within clinical practice contexts (Piantadosi, 2024), including the development of optimal AI-assisted diagnostic processes, and assessing their impact on diagnostic accuracy, treatment decisions, and patient prognosis, will be imperative. Overall, future research will focus on dataset expansion, multicenter validation, model optimization, interpretability enhancement, and clinical application research to advance the comprehensive application of AI technology in liver cancer diagnosis.

## 5 Conclusion

In this study, an efficient and accurate deep learning model, HTRecNet, was successfully developed for the early diagnosis of liver cancer. Through the incorporation of the SPConv module and the CBAM attention mechanism, an innovative enhancement of the ResNet50 benchmark model was achieved, improving diagnostic accuracy while significantly reducing computational complexity. Experimental results demonstrate that HTRecNet excels in classifying HCC, CCA, and normal liver tissues, showcasing exceptional generalization ability on external independent test datasets. The visualization of heat maps further validates the model’s feature extraction capability and the interpretability of the diagnostic process, providing crucial technical support for clinical applications. However, the study faces some limitations, such as an unbalanced dataset and a restricted external validation dataset. Future research endeavors will prioritize expanding the dataset size, conducting multi-center validation, optimizing the model structure, enhancing interpretability, and advancing clinical applications to further bolster the performance and practical application value of HTRecNet. In conclusion, the outstanding performance and potential application value of HTRecNet in liver cancer diagnosis have established a robust foundation for future research and practical clinical implementations.

## Data Availability

The original contributions presented in the study are included in the article/supplementary material, further inquiries can be directed to the corresponding authors.
